# Supplementary material for: Comprehensive Metabolomics and Machine Learning Identify Profound Oxidative Stress and Inflammation Signatures in Hypertensive Patients with Obstructive Sleep Apnea
Source: Antioxidants (Basel). 2022 Sep 29;11(10):1946. doi: 10.3390/antiox11101946 (PMC9598902; doi:10.3390/antiox11101946)
Supplement: Supplementary file 1 [file antioxidants-11-01946-s001.zip › antioxidants-1916426-supplementary.pdf]

antioxidants

Supplementary material

Method

Detailed approaches of serum metabolome and lipidome analyses

The hydrophilic residues were suspended with 150 µL of acetonitrile: water (1:1, v/v) solution and performed on a Acquity UPLC HSS T3 C18 column (100 × 2.1 mm, 2.5 µm; Waters Corp., Milford, MA, USA). The optimal mobile phase consisted of a linear gradient system of phase A (0.1% formic acid in water), and mobile phase B (0.1% formic acid in acetonitrile): 0 min, 100% A; 2.5 min, 85% A; 10 min, 55% A; 12 min, 50% A; 14.5 min, 0% A; 15.5 min, 0% A; and 17.5 min, 100% A. The lipid residues were reconstituted in 150 µL of chloroform: methanol (1:1, v/v) solution and then diluted threefold with isopropanol: acetonitrile: water (2:1:1, v/v/v) solution. The separation of lipids was performed on a reversed phase X-select CSH C18 column (2.1 mm × 100 mm, 2.5 µm; Waters Corp., Milford, MA, USA). The mobile phase consisted of a linear gradient system of phase A (10 mM ammonium acetate and 0.1% formic acid in acetonitrile with 40% water), and mobile phase B (isopropanol: acetonitrile, 9:1, v/v): 0 min 60% A; 2 min 57% A; 12 min 40% A; 12.1 min 25% A; 18 min 1% A; 19 min 1% A; 20 min 40% A. The sample injection volume of each sample was 10 µL. The column temperature was maintained at 40 °C and the flow rate was 0.4 mL/min. Mass spectrometry (MS) analyses were performed on the Q-Exactive HF MS (Thermo Fisher Scientific, Waltham, MA, USA) by using data-dependent acquisition model. The spray voltage was 3.3 kV for positive ion mode and 2.5 kV for negative ion model. Each acquisition cycle consists of one survey scan at 60,000 resolutions from 50 to 900 m/z for the hydrophilic metabolites and 150 to 1200 m/z for lipids, followed by 10 MS/MS scans at 15,000 resolutions using step-NCE of 15, 30 and 45. The dynamic exclusion was set to 10 s, sheath gas was 40 L/h; aux gas was 10 L/h. The probe heater temperature and capillary temperature were set as 300 °C and 320 °C, respectively. To monitor the robustness of sample pretreatment and stability of LC-MS analysis, the quality control (QC) samples were analyzed between every ten test samples.

All the raw data was analyzed by using MS-DIAL software v3.6 for deconvolution, alignment, and data reduction to provide a comprehensive data matrix, including information of precursor ions, fragment ions, neutral molecules, retention times, and raw intensity. Metabolite identification was performed on in MS-DIAL and Progenesis QI software (Waters, Manchester, U.K.) by comparing the exact molecular mass and fragments using QI MetaScope, HMDB, METLIN, and LIPIDMAPS databases. The matched metabolite was further identified by the isotopic distribution measurement and the retention times and MS/MS fragmentation patterns of authentic standards using in-house metabolite library. Then, the raw data matrix was normalized by the QC samples and the intensity of internal standards. Metabolome and lipidome datasets were merged for subsequent statistical analysis.

Appendix B

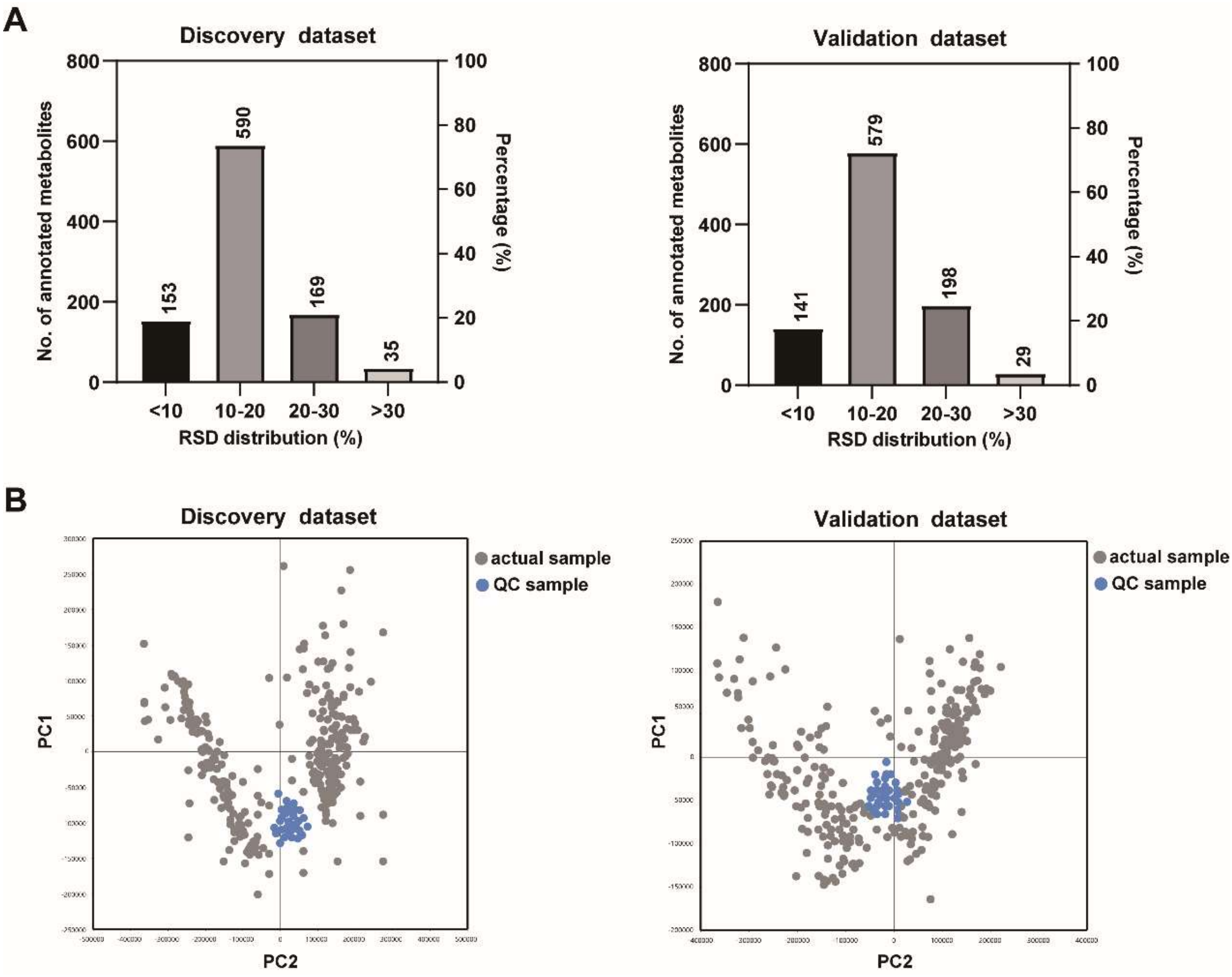

Figure S1. Stability evaluation of metabolomic data. (A) Stability of analytical methods based on QC samples. (B) PCA scores plot of test samples and QC samples; each point represents an individual serum sample.

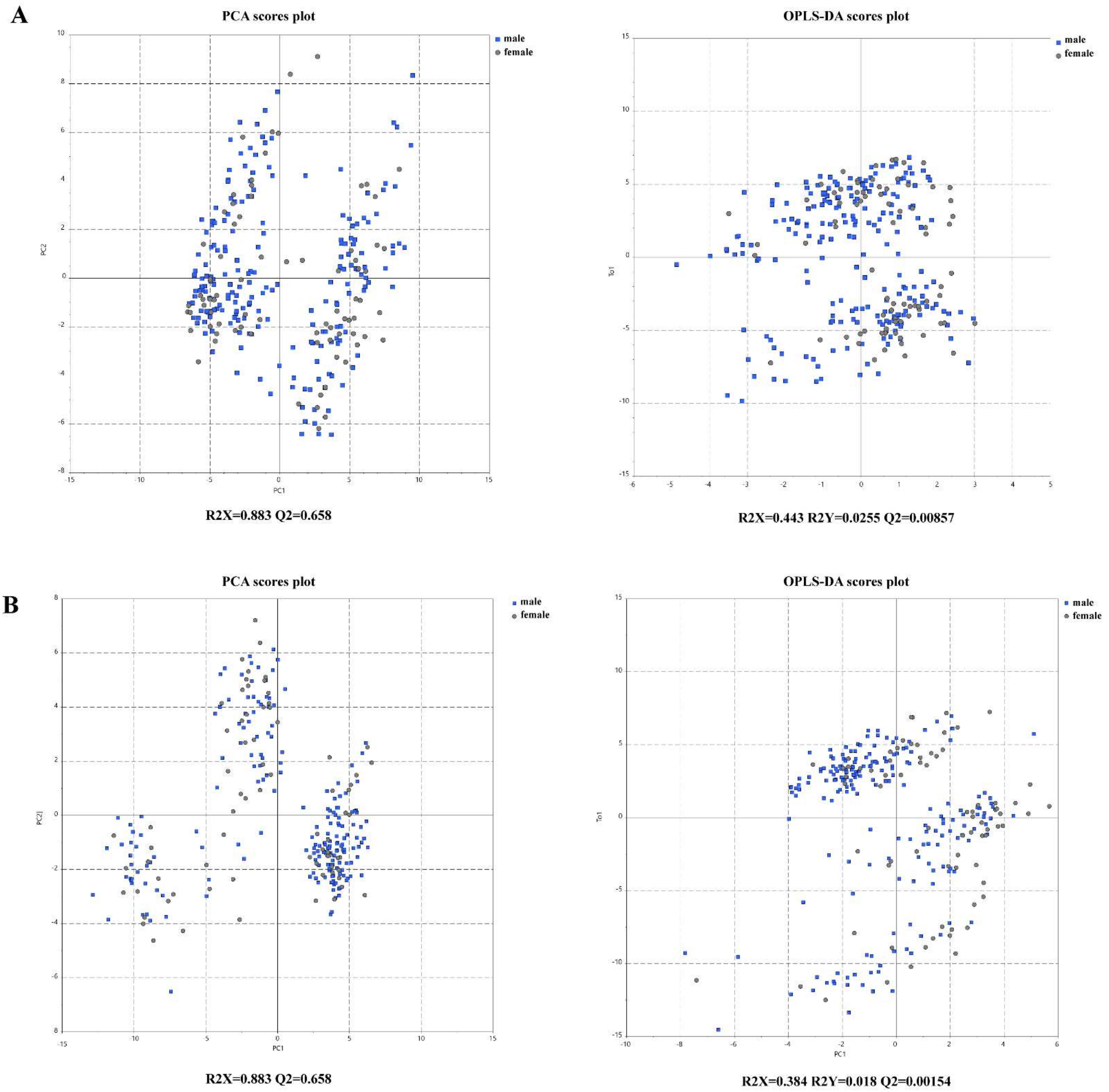

Figure S2. Multivariate statistical analyses of the correlation between discriminatory metabolite signatures and gender. (A**)** unsupervised principal component analysis (PCA) and supervised orthogonal partial least square discriminant analysis (OPLS-DA) score plots of discriminatory metabolite signatures from discovery set showing no variance due to gender. (B) PCA and OPLS-DA score plots of discriminatory metabolite signatures from validation set showing no variance due to gender.

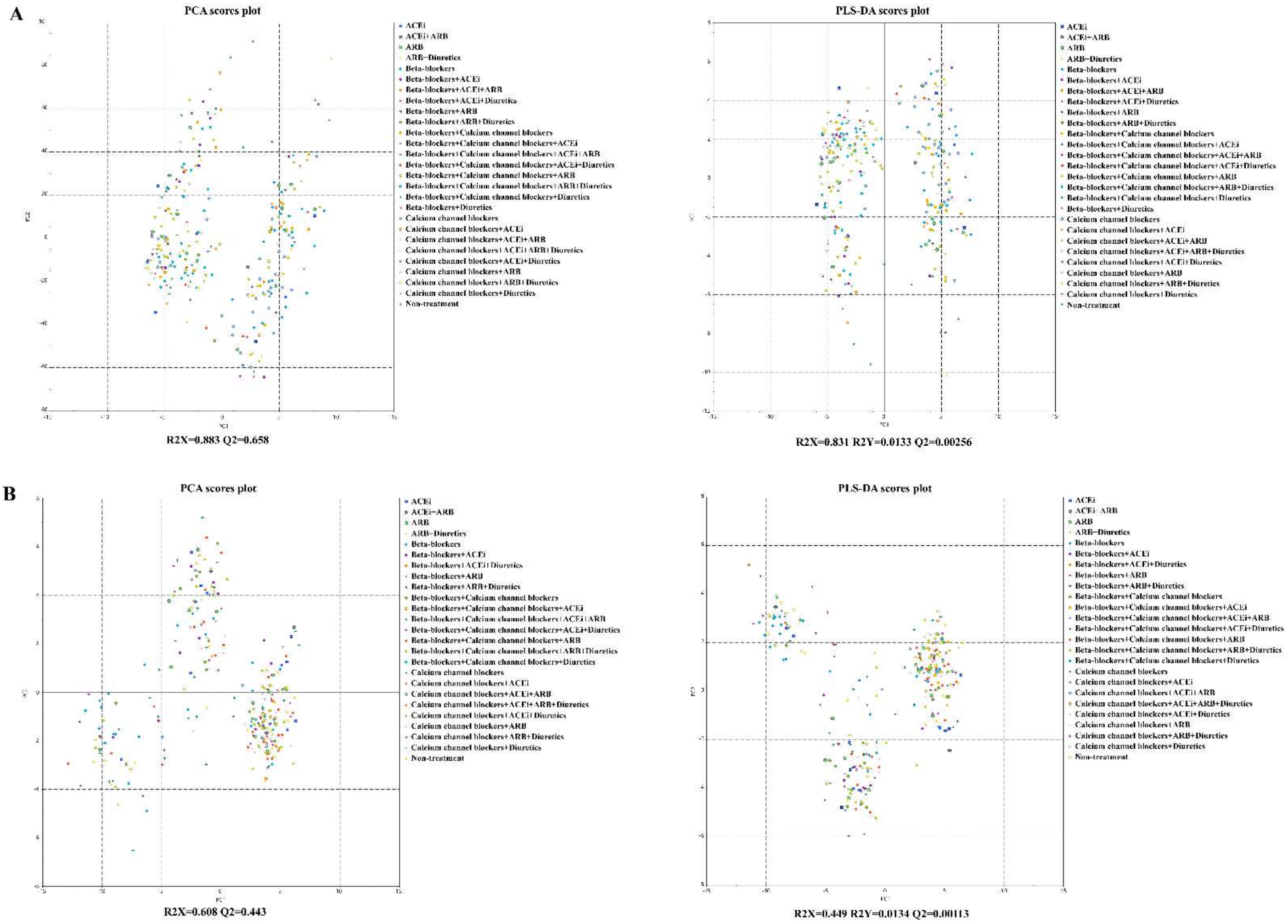

Figure S3. Multivariate statistical analyses of the correlation between discriminatory metabolite signatures and antihypertensive drugs. (A**)** unsupervised PCA and supervised partial least square discriminant analysis (PLS-DA) score plots of discriminatory metabolite signatures from discovery set showing no variance due to different antihypertensive drugs and their combination. (B) PCA and PLS-DA score plots of discriminatory metabolite signatures from validation set showing no variance due to different antihypertensive drugs and their combination.

Table S1 Differentiated metabolites in the serum samples from OSA and non-OSA patients.

ID	Discovery: OSA vs. non-OSA			Validation: OSA vs. non-OSA		
	$\log_2$ (fold change)	P value	FDR P value	$\log_2$ (fold change)	P value	FDR P value
12-HEPE	1.41	2.66E-13	2.87E-13	1.56	2.26E-30	1.29E-29
13-HODE	1.47	4.78E-13	5.1E-13	0.55	2.93E-08	3.84E-08
12-HETE	1.72	3.03E-56	1.1E-55	1.35	4.22E-34	3.76E-33
5-HETE	1.43	1.18E-20	1.45E-20	1.85	4.94E-36	6.58E-35
2-Oxobutyric acid	-1.72	4.17E-48	9.03E-48	-0.78	1.72E-09	2.46E-09
3-Hydroxy-3-methylglutaric acid	-1.71	4.33E-67	2.47E-66	-0.81	6.09E-08	7.85E-08
2-Hydroxyglutaric acid	-2.20	1.15E-54	3.83E-54	-1.44	1.43E-28	6.01E-28
3-Hydroxyphenylacetic acid	-2.11	3.83E-42	7.65E-42	-1.86	2.32E-36	3.71E-35
3-Methoxytyrosine	-2.74	1.88E-73	1.16E-72	-1.21	6.17E-09	8.66E-09
5-Hydroxytryptophan	0.84	1.37E-10	1.41E-10	1.32	1.79E-21	4.94E-21
6-Hydroxycaproic acid	-2.50	4.76E-65	2.5E-64	-1.04	7.99E-21	2.06E-20
Acetylcarnitine	-1.28	3.89E-24	5.28E-24	-1.27	2.08E-23	6.18E-23
All-trans-Retinoic acid	0.80	5.37E-17	6.05E-17	0.89	1.24E-16	2.48E-16
Arachidonic acid	0.99	8.35E-22	1.08E-21	0.82	2.6E-21	6.93E-21
Arginine	-1.17	5.49E-23	7.2E-23	-1.35	2.66E-33	2.13E-32
Aspartic acid	0.72	6.29E-09	6.37E-09	0.94	2.54E-14	4.72E-14
Citric acid	-1.64	1.98E-37	3.59E-37	-0.68	5.81E-05	6.64E-05
CAR 18:0	1.07	1.98E-39	3.69E-39	1.03	2.04E-29	9.58E-29
Choline	0.37	9.84E-21	1.23E-20	0.23	4.07E-07	4.86E-07
DAG 32:0	1.98	4.68E-28	6.46E-28	1.34	2.6E-25	8.67E-25
DAG 34:2	1.72	9.5E-37	1.62E-36	1.36	2.09E-30	1.29E-29
D-Glucuronic acid	-1.72	2.81E-31	4.17E-31	-1.14	1.68E-18	3.85E-18
Dihydrosphingosine	3.65	1.57E-90	4.2E-89	3.18	9.23E-30	4.92E-29
Dopamine	0.92	4.35E-19	5.19E-19	0.42	2.23E-07	2.75E-07
Glycylproline	-1.52	9.58E-51	2.25E-50	-0.95	2.6E-10	4E-10
Hypotaurine	2.19	1.26E-52	3.37E-52	2.56	7.96E-38	2.12E-36
Indole	1.27	1.1E-32	1.73E-32	0.35	7.68E-05	8.66E-05
Indoleacetic acid	1.05	4.57E-15	5.07E-15	0.56	2.97E-07	3.6E-07
Glutamine	-1.31	1.94E-31	2.93E-31	-1.35	5.76E-29	2.56E-28
Ketoleucine	-0.35	0.000455	0.000455	-0.52	1.59E-07	1.99E-07
Pyroglutamic acid	-1.70	2.41E-62	1.02E-61	-1.42	1.76E-38	7.03E-37
L-alanine	-1.51	2.29E-47	4.81E-47	-0.57	6.39E-06	7.52E-06
L-Carnitine	0.93	2.73E-34	4.37E-34	0.70	7.24E-27	2.52E-26
Leucylproline	1.24	1.4E-43	2.88E-43	0.75	1.29E-11	2.19E-11
L-Histidine	-1.07	3.76E-32	5.78E-32	-1.35	3.43E-34	3.43E-33

LPA 16:0	1.60	1.74E-64	7.73E-64	1.13	1.39E-36	2.78E-35
LPC 16:0	1.49	1.4E-143	1.1E-141	0.66	2.82E-35	3.22E-34
LPC 16:1	1.26	2.31E-54	7.4E-54	0.62	5.33E-17	1.12E-16
LPC 18:0	1.63	1.67E-86	2.22E-85	0.74	4.62E-20	1.16E-19
LPC 18:1	1.04	2.31E-78	1.85E-77	0.45	3.17E-13	5.63E-13
LPC 18:2	1.27	4.5E-122	1.8E-120	0.57	1.34E-24	4.13E-24
LPC 20:2	1.49	7.86E-51	1.91E-50	1.01	1.38E-27	5.03E-27
LPC 20:4	1.33	5.01E-65	2.5E-64	0.46	1.44E-11	2.35E-11
Malic acid	-1.03	5.77E-65	2.72E-64	-0.80	1.07E-19	2.59E-19
Methionine	-1.91	3.36E-87	5.38E-86	-0.97	4.76E-14	8.66E-14
Methoxytyramine	-1.29	1.48E-48	3.28E-48	-1.07	1.76E-18	3.91E-18
N8-Acetylspermidine	1.55	5.39E-60	2.16E-59	0.90	2.45E-31	1.64E-30
Norvaline	-1.22	5.75E-35	9.38E-35	-0.74	8.31E-19	1.96E-18
Oxypurinol	2.13	1.06E-52	2.92E-52	0.99	9.7E-28	3.88E-27
Palmitic acid	0.74	7.35E-13	7.73E-13	0.26	0.020299	0.021652
PC 34:2	1.29	4.82E-40	9.41E-40	1.30	1.09E-29	5.45E-29
PC 35:5	2.97	5.62E-77	4.09E-76	1.25	6.69E-25	2.14E-24
Phenylalanine	-0.58	1.08E-17	1.26E-17	-0.68	1.36E-15	2.65E-15
Proline	-1.46	1.52E-51	3.79E-51	-0.68	1.59E-08	2.15E-08
Prolylhydroxyproline	-2.27	2.78E-75	1.85E-74	-1.01	3.44E-10	5.2E-10
Purine	-2.10	1.14E-79	1.02E-78	-1.03	7.05E-11	1.11E-10
Sarcosine	-1.54	3.68E-53	1.05E-52	-0.66	1.42E-11	2.35E-11
Stearic acid	0.73	3.94E-21	5.01E-21	0.32	1.38E-07	1.76E-07
Taurine	-1.60	6.65E-29	9.5E-29	-1.72	4.17E-32	3.03E-31
Taurocholic acid	1.33	2E-55	6.94E-55	0.53	1.52E-15	2.9E-15
Tyrosine	2.04	1.28E-87	2.55E-86	0.75	5.37E-22	1.53E-21
Uridine	-0.90	6.14E-37	1.07E-36	-0.33	0.000427	0.000475
Linoleic acid	1.00	2.17E-13	2.38E-13	0.26	0.01023	0.011059

Two-tailed Student's *t* test or Mann Whitney *U* test were used for each comparison. $\log_2$ (fold change) > 0 and <0 indicates up-regulated and down regulated in OSA compared to non-OSA. FDR = false discovery rate.
